# Adsorptive removal of noxious cadmium from aqueous solutions using poly urea-formaldehyde: A novel polymer adsorbent

**DOI:** 10.1016/j.mex.2018.09.010

**Published:** 2018-09-29

**Authors:** Mohammad Hadi Dehghani, Samira Tajik, Ahmad Panahi, Mostafa Khezri, Ahmad Zarei, Zoha Heidarinejad, Mahmood Yousefi

**Affiliations:** a Department of Environmental Health Engineering, School of Public Health, Tehran University of Medical Sciences, Tehran, Iran; b Institute for Environmental research, Center for Solid Waste Research, Tehran University of Medical Sciences, Tehran, Iran; c Department of Environmental Engineering, Science and Research Branch, Islamic Azad University, Iran; d Department of Environmental Health Engineering, Faculty of Health, Gonabad University of Medical Sciences, Gonabad, Iran; e Department of Environmental Health Engineering , Faculty of Health , Hormozgan University of Medical Sciences, Bandar Abbas, Iran

**Keywords:** Poly urea-formaldehyde, Cadmium, Adsorption, Aqueous solutions

## Abstract

Cadmium is a heavy metal toxic that enters water resources through industrial, household, agricultural waste and non-sanitary landfill of urban and industrial wastes. Pollution of water resources by cadmium increases incidence of diseases including Itai-Itai, kidney disorders, cancer, chromosome effects and kidney tubular damages in low exposures. The aim of this study is to study the efficiency of a new poly urea-formaldehyde adsorbent in the removal cadmium ions from aqueous solutions. The effect of different variables such as initial pH, contact time, initial concentration of cadmium and test of real wastewater samples were evaluated. In addition, laboratory data of cadmium adsorption by urea-formaldehyde adsorbent were matched to Langmuir, Freundlich and Temkin isotherm models. The results of the study showed that maximum adsorption capacity obtained by Langmuir model was 76.3 mg/g at pH = 5.5. Laboratory adsorption data matched mostly by Freundlich isotherm model (R^2^ =0.999) which indicates that adsorption of cadmium ions on heterogenic surfaces of poly urea-formaldehyde happens by chemical adsorption mechanism. Generally, the results of the study showed that new poly urea-formaldehyde adsorbent can be efficiently used to remove highly concentrated cadmium ions from aqueous solutions.

## Graphical abstract

**Figure:**
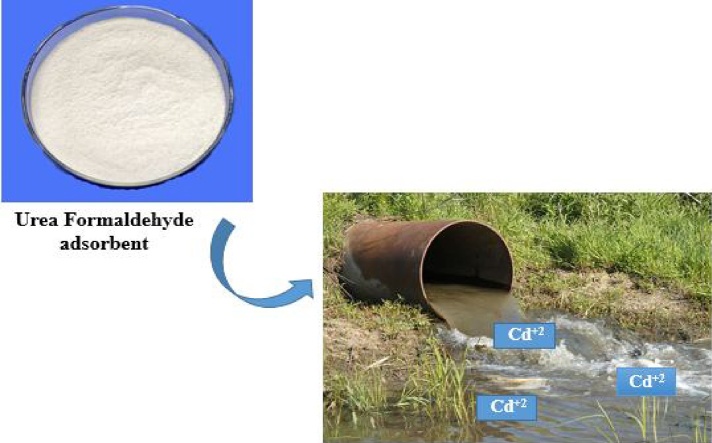

Specifications Table

**Table:** 

Subject area	Environmental Chemical Engineering
• More specific subject area	• Adsorption
• Protocol name	• Application of new urea formaldehyde adsorbent in the removal of cadmium from aqueous solutions.
• Reagents/tools	• The Cd^+2^ concentration measurement was performed by an Atomic Absorption Spectrophotometer (Shimadzu AA- 670 model). • A digital pH meter (Basic 20 Crison) was used for solution pH measurement.
• Experimental design	• Measuring of Cd^+2^ concentrations under various levels of initial Cd^+2^ concentration, solution pH, and contact time to obtain optimal Cd^+2^ removal from aqueous solution using a novel adsorbent provided from Urea Formaldehyde.
• Trial registration	• No applicable
• Ethics	• No applicable

## Protocol data

• Preparation a new highly efficient polymer urea-formaldehyde for the removal of cadmium ions from aqueous solutions.
• Maximum adsorption capacity of cadmium was 76.3 mg/g.
• Suitable application of urea-formaldehyde adsorbent in removing cadmium ions from wastewater is shown.

## Description of protocol

### Chemicals and materials

All chemical materials used in the present research were in analytical grade. Chemicals including cadmium nitrate, urea, formaldehyde, acetic acid (CH_3_COOH) (purity degree of 100%), nitric acid (purity degree of 65%), acetate sodium (C_2_H_3_NaO_2_), sodium dihydrogen phosphate (NaH_2_PO_4),_ di-sodium hydrogen phosphate (Na_2_HPO_4_) and sodium hydroxide (NaOH) were provided from Merck company, Germany. A stock solution of 500 mg/L of cadmium was prepared by dissolving a certain amount of cadmium nitrate salt in de-ionized water. Different concentrations of cadmium solution were prepared by diluting the stock solution.

### Urea-formaldehyde preparation

**Fig. 1:**
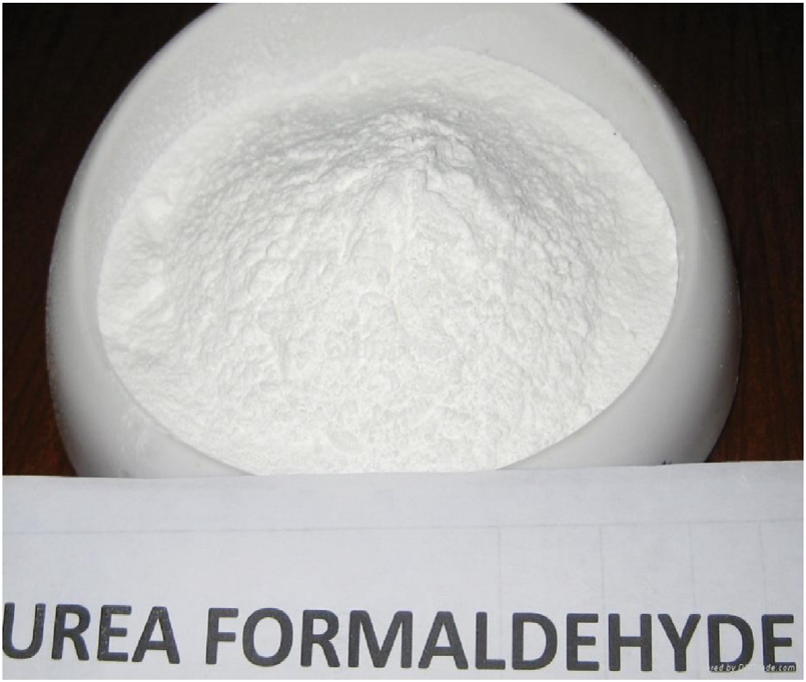
Urea Formaldehyde.

**Fig. 2:**
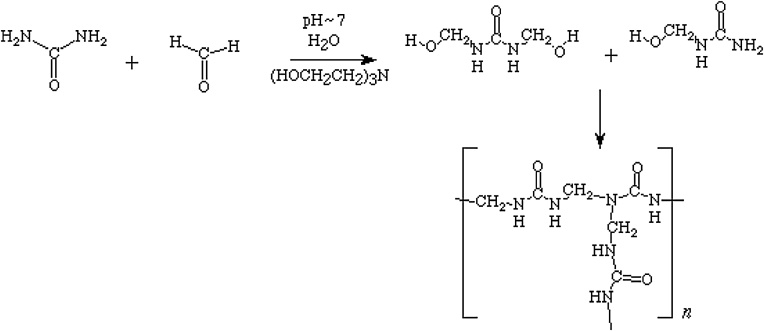
The mechanism of urea and formaldehyde reactions.

In order to prepare urea-formaldehyde adsorbent, firstly, 6 mL of formaldehyde was poured into volumetric flask and NaOH 0.1 M was added to it drop by drop for pH of the solution reach to 8. Then, 3 gr of urea was added to the solution and it was kept in a 50 °C hot water bath to be dissolved uniformly. After that, the solution was kept at 70 °C to be completely colorless. In the next step, 2 mL of cadmium with a concentration of 500 mg/L was added to the obtained solution and then 0.5 mL acetic acid 100% was added to it. After some time, obtained solution was stiff as a polymer. Then, it was ground as a powder. Then the polymer was washed three times by 50 mL nitric acid 0.1 M, each time for half an hour and the solution pH was adjusted to 6. Then the polymer was percolated and dried to remove any moisture. Finally, prepared adsorbent was used to perform cadmium adsorption tests (Fig. 1, Fig. 2).

### Adsorption tests

All cadmium adsorption tests on urea formaldehyde polymeric adsorbent were performed using 50 mL of cadmium solution in 100 mL Erlenmeyer at a temperature range 22–24 °C on a 300 rpm shaker. In this study, the effects of initial pH (3.5, 4.5, 5.5, 6.5, 7.5, 8.5), contact time (1, 2, 5, 10, 20, 30, 60, 90 min) and initial cadmium concentrations (5, 10, 20, 40, 60, 80 mg/L) parameters were studied. Before adsorption process, in order to prepare buffers with pH ranging from 3.5 to 6.5, acetate sodium (C_2_H_3_NaO_2_) 0.01 M and acetic acid 0.01 M (CH_3_COOH) were used and to prepare buffers with a pH ranging from 6.5 to 8.5, sodium dihydrogen phosphate (NaH_2_PO_4)_ 0.01 M was used. pH of the solutions was measured by a pH-meter (model Basic 20 Crison). After adsorption tests, adsorbents were removed from solution by membrane filter and the concentration of filtered cadmium solution was measured by an Atomic Absorption Spectrophotometer (Shimadzu AA-670 model) equipped with GFA-4B graphite furnace atomizer and D2 lamp for background correction. Cadmium hollow cathode lamp was applied as radiation source at 4 mA. An atomic absorption signal at 228.8 nm line was recorded on a graphic printed PR-4 with peak height and gas stop mode for quantification.

### Cadmium removal in real samples

Efficiency of urea formaldehyde adsorbent for removal of cadmium from real sample (wastewater) was also studied. The sample was collected from effluent of a wastewater treatment plant located in south of Tehran in Ray. First, concentration of cadmium ion was measured in real sample which was 0.078 mg/L. Then, 2 mg/L cadmium was added to the real sample and they were put next to 1 gr adsorbent for 15 min under optimum pH conditions at 22–24 °C and the amount of cadmium adsorption was determined by atomic adsorption spectroscopy method. To improve accuracy and validity, all adsorption tests were repeated in triple.

## Data analysis

In surveying efficiency of synthesized polymer on real samples equations (1) and (2) were used to calculate percentage of standard deviation and relative standard deviation, respectively:

(1) $$SD=\frac{\sqrt{\sum {\left(x-\bar{x}\right)}^{2}}}{n-1}$$

(2) $$RSD\left(\%\right)=\frac{SD}{\bar{x}}\times 100$$

X: Residual concentration after adsorption process in the solution in mg/L

$\bar{X}:$ Mean residual concentration after adsorption process in mg/L

SD: Standard deviation

RSD (%): Relative standard deviation

N: Number of times that test performed

In order to calculate the efficiency of cadmium removal by urea-formaldehyde equation (3) was used [[1], [2], [3]].

(3) $$RE=\frac{\left(C_{i}-C_{t}\right)}{C_{i}}\times 100$$

Where, RE is removal efficiency (%), C_i_ is initial concentration of pollutant in the solution (mg/L) and C_t_ is final concentration of pollutant in the solution (mg/L).

## Effect of pH

**Fig. 3:**
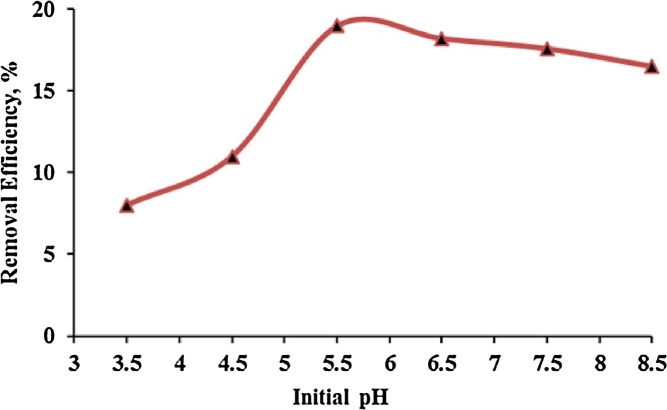
The effect of pH on cadmium adsorption by urea formaldehyde.

Solution pH is one of the most important parameters in adsorption process, because the binding of cations to active surface groups is strongly dependent on the surface charge of particles [[4], [5], [6]]. The effect of pH on the rate of adsorption of cadmium on urea formaldehyde adsorbent is shown in Fig. 3. In this research, after testing different pHs, according to Fig. 3 highest adsorption efficiency was obtained for cadmium ion by formaldehyde adsorbent at pH = 5.5 which indicates that this adsorbent acts better in weak acidic and near to neutral pH and has higher adsorption in this condition, while at pHs lower than 5.5, adsorption rate decreases and this is because at lower pHs, there is competition between H^+^ ions and cadmium ions, but in pHs higher than 5.5 the rate of cadmium adsorption by studied adsorbent increases and this may be due to the formation of solution complexes [[7], [8], [9]].

## Effect of contact time

**Fig. 4:**
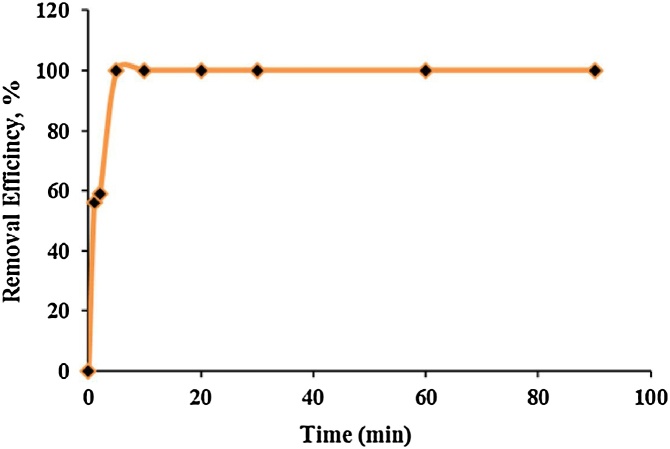
The effect of contact time on cadmium adsorption by urea formaldehyde.

Contact time between adsorbate and adsorbent is an important parameter in adsorption process [[10], [11], [12]]. As shown in Fig. 4, during first 5 min shaking, highest cadmium adsorption has happened by urea formaldehyde and by passing time, adsorption efficiency did not considerably change and was mostly stable until the adsorbent was saturated. This can be attributed to the availability of more vacant biding sites for cadmium adsorption during initial contact times [6].

## Adsorption isotherms

In order to design an adsorption process for application in field, the isotherms for an adsorbate-adsorbent system should be determined experimentally [13]. The isotherms show the chemical equilibrium for the specific conditions of temperature, pH, adsorbent dosage etc. and thus indicate the maximum achievable adsorption capacity in adsorption process. To study the isotherm of cadmium adsorption by urea formaldehyde polymer, Langmuir, Freundlich and Temkin adsorption isotherm models were used in optimum conditions.

### Langmuir isotherm

Langmuir isotherm is valid for single layer surface adsorption and was first performed with the aim of describing gas surface adsorption on activated carbon [14].

Assumptions of Langmuir model: 1-adsorption energy is the same and does not depend on the amount of absorbed material on the adsorbent, in other words, the adsorption capability of each site is the same as the other and the presence of absorbed material in each site has no other effect, 2-adsorption bonds are reversible and 3-absorbed material is a layer with thickness of a molecule [15,16].

The linear form of Langmuir isotherm is represented by the expression:

(4) Ceqe=Ceqmax+1K qLmax

Where, $q_{e}$ is the amount of contaminant per 1 gr of adsorbent, $C_{e}$ is the amount of remaining contaminant after equilibrium, $q_{\max}$ is maximum amount of absorbed substance after equilibrium and b is Langmuir constant.

### Freundlich isotherm

The most important multisite adsorption isotherm for heterogeneous surfaces is the Freundlich adsorption isotherm [17]. Freundlich isotherm indicates non ideal, reversible and multilayer adsorption with heterogeneous distribution of heat and adsorption on heterogeneous surface [18]. The non-linearized form of Freundlich isotherm model is given in the following equation:

(5) $$q_{e}=k_{F}C_{e}^{1/nf}$$

Freundlich isotherm can also be written as follows:

(6) $$1\text{n}\;q=\text{1n}\;\mathrm{K+}\frac{1}{n}1n\;C_{f}$$

LnK is y-intercept. Slope of this line is $\frac{1}{n}$ which indicates adsorption intensity and K indicates adsorption capacity.

### Temkin adsorption isotherm

The Temkin isotherm is usually applied for heterogeneous surface energy systems (non-uniform distribution of sorption heat) [19]. Assumptions of Temkin model: 1- adsorption is exponential, and 2- adsorption is single layer. Temkin isotherm is presented as:

(7) q_e =_ BLnA + B LnC_e_

In the above equation, B = RT/b is a constant showing heat of sorption (J/mol) obtained from the Temkin plot (qe against lnCe), A (slope) is Temkin isotherm equilibrium binding constant (L/g), b (intercept) is Temkin isotherm constant, R is universal gas constant (8.314 J/mol.k) and T is absolute temperature (Kelvin).

**Fig. 5:**
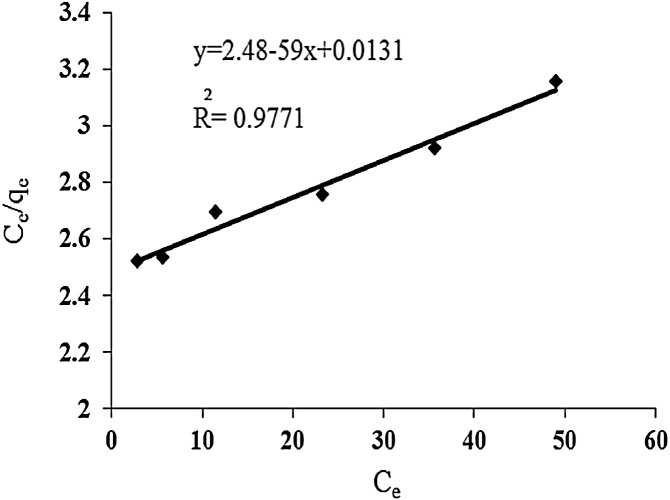
Langmuir isotherm for cadmium adsorption.

**Fig. 6:**
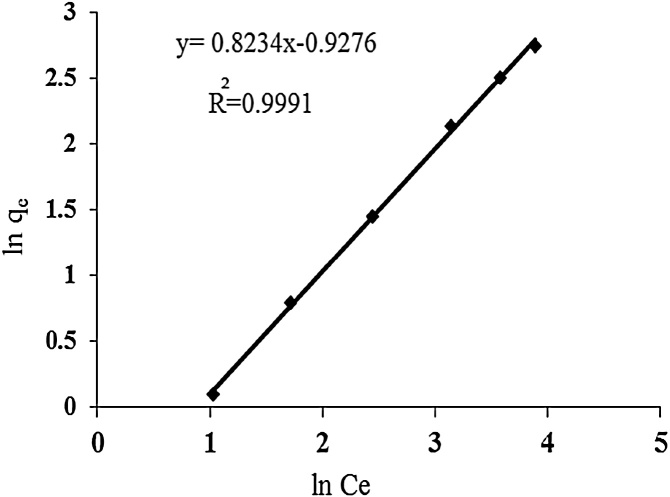
Freundlich isotherm for cadmium adsorption.

**Fig. 7:**
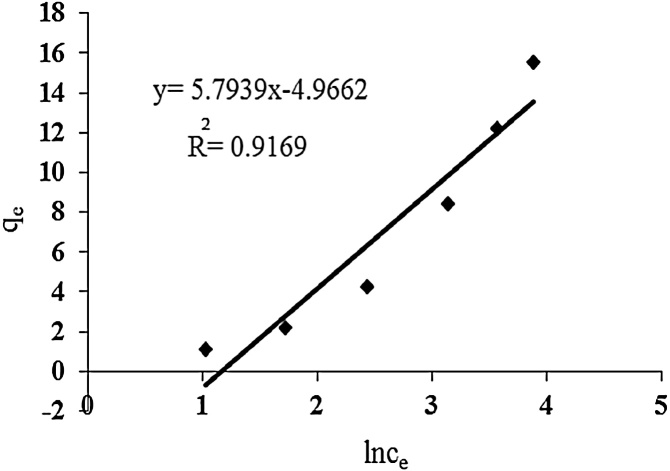
Temkin isotherm for cadmium adsorption.

In this study, three isotherm models such as Langmuir, Freundlich, and Temkin adsorption isotherms were used. Maximum adsorption capacity was 76.3 mg/g. Freundlich, Langmuir and Temkin adsorption isotherms parameters are presented in Table 1. Considering R^2^ correlation coefficient, the results showed that cadmium adsorption on urea formaldehyde adsorbent best matches Freundlich isotherm model. Since Freundlich isotherm indicates multi-layer adsorption on heterogeneous surfaces, it can be concluded that cadmium adsorption by urea formaldehyde occurred in multi layers (Fig. 5, Fig. 6, Fig. 7).

**Table 1:** Parameters and correlation coefficients of isotherm models of cadmium adsorption on urea formaldehyde.

Isotherm	Isotherm formula	Parameters	Values
Langmuir	Ceqe=Ceqmax+1K qLmaxRL=1/1+KLC0	q_max (mg/g)_	76.3
		K_L (lit/g)_	0.005
		R^2^	0.97
Freundlich	Lnq_e=_lnK_F_+1/n LnC_e_	K_F_	0.44
		n	1.078
		R^2^	0.99
Temkin	${\text{q}}_{\text{e}}=\begin{array}{c} \text{BLnA}+\text{B}\;\text{Ln}{\text{C}}_{\text{e}}\\ \text{B}=\text{RT}/\text{b} \end{array}$	A	0.3116
		B	4.966
		R^2^	0.91

## Study of cadmium removal in real samples

Results of tests performed on real sample are presented in Table 2. Regarding the test performed on considered adsorbent, the value of standard deviation was obtained 2.47% that indicates low errors of tests. Considering Table 2, the rate of cadmium removal on real wastewater samples by urea formaldehyde adsorbent is equal to 54.6% that indicates good function of adsorbent in absorbing cadmium from aqueous solutions.

**Table 2:** Standard deviation and relative standard deviation of cadmium ion adsorption in the real samples in 3 steps.

Initial concentration of cadmium (mg/L)	Added concentration of cadmium (mg/L)	Total concentration of cadmium (mg/L)	Cadmium concentration after adsorption (mg/L)	Mean concentration (mg/L)	Rate of adsorption (g)	SD	RSD (%)	Removal percentage
0.078	2	2.078	0.924	0.946	1	0.0234	2.47	54.5
			0.940					
			0.975					
